# Obstructive Sleep Apnea Syndrome: From Phenotype to Genetic Basis

**DOI:** 10.2174/138920209787846998

**Published:** 2009-04

**Authors:** M Casale, M Pappacena, V Rinaldi, F Bressi, P Baptista, F Salvinelli

**Affiliations:** 1 Area of Otolaryngology, University Campus Bio-Medico, Rome, Italy; 2 Neurology, University Campus Bio-Medico, Rome, Italy; 3 Departamento de Otorrinolaringología, Clínica Universitaria de Navarra, Pamplona, Navarra, Spain

**Keywords:** Obstructive sleep apnea, genetic, hypopnea, AHI, snoring, risk factors, phenotype, multifactorial disease.

## Abstract

Obstructive sleep apnea syndrome (OSAS) is a complex chronic clinical syndrome, characterized by snoring, periodic apnea, hypoxemia during sleep, and daytime hypersomnolence. It affects 4-5% of the general population. Racial studies and chromosomal mapping, familial studies and twin studies have provided evidence for the possible link between the OSAS and genetic factors and also most of the risk factors involved in the pathogenesis of OSAS are largely genetically determined. A percentage of 35-40% of its variance can be attributed to genetic factors. It is likely that genetic factors associated with craniofacial structure, body fat distribution and neural control of the upper airway muscles interact to produce the OSAS phenotype. Although the role of specific genes that influence the development of OSAS has not yet been identified, current researches, especially in animal model, suggest that several genetic systems may be important. In this chapter, we will first define the OSAS phenotype, the pathogenesis and the risk factors involved in the OSAS that may be inherited, then, we will review the current progress in the genetics of OSAS and suggest a few future perspectives in the development of therapeutic agents for this complex disease entity.

## INTRODUCTION

### Definition

The obstructive sleep apnea syndrome (OSAS) is a potentially disabling condition, characterized by excessive daytime sleepiness, disruptive snoring, recurrent episodes of apnea (no airflow) or hypopnea (partially obstructed airflow), and nocturnal hypoxemia, generally defined as five or more apneas–hypopneas per hour of sleep (i.e., the apnea–hypopnoea index-AHI).

OSAS is usually associated with a history of habitual snoring, which is a sign of increased pharyngeal airflow resistance. An OSAS is defined as an AHI, equal to or more than 5 accompanied by either excessive daytime sleepiness or two or more of episodes of choking or gasping during sleep, recurrent awakenings, unrefreshing sleep, daytime fatigue, or impaired concentration or memory [1]. The American Academy of Sleep Medicine classification of OSAS severity considers both the AHI (mild defined as an AHI of 5–15, moderate as 15–30, and severe as >30) and the degree of daytime sleepiness (mild: unwanted sleepiness or involuntary sleep episodes occurring during activities that need little attention; moderate: during activities that need some attention, such as during meetings; severe: during activities that need more active attention such as during conversation or driving). However, these thresholds are arbitrary [2].

OSAS must be considered as a multifactorial disease. Multiple genes, environmental influences, and development factors are closely related to OSAS. Redline and Tishler [3] emphasize on the genetic basis of several risk factors for obstructive sleep apnea. These risk factors include obesity, central control of ventilation, and craniofacial morphology. Probably for this reason, symptoms of sleep apnea syndrome have been found to be more common in the families of patients with this syndrome.

### Epidemiology

OSAS was first identified only 40 years ago and its clinical importance is increasingly recognized. Although now acknowledged as a worldwide problem, which affects 2-4% of middle-aged men and 1-2% of middle-aged women in Western countries, the majority of affected individuals remain undiagnosed. OSAS is strongly associated with obesity currently occurring in many countries, but is also increasingly identified in normal subjects, in whom a particular craniofacial structure is an important contributory factor [4, 5].

OSAS is two to three-times more common in men than in women, and in those aged 65 years or more than in those aged 30–64 years.

In postmenopausal women, the OSAS prevalence tends to increase, particularly in women without hormone replacement therapy, but it remains lower than men of the same age stratum [6]. Although OSAS mainly affects adult subjects, its presence in children should not be neglected. This is not only because of its relatively high prevalence (2% in children aged 2–8 years, apparently related to adenotonsillar hypertrophy), but also because of its clinical consequences, including hypertension, nocturnal enuresis, growth retardation, cognitive impairment, and hyperactivity [7]. Excessive daytime sleepiness is reported to be associated with a higher risk of motor vehicle accidents and work place injuries or poor work performance [8].

### Diagnosis of OSAS

#### Clinical Picture

OSAS is characterized by several signs and symptoms. All of these can be divided into “nocturnal” and “daytime” symptoms.

#### Nocturnal Symptoms

##### Snoring:

Snoring is the most frequent symptom of OSAS. It is caused by the narrowing of the upper airway and occurs in up to 95% of patients. Anyway, it has poor predictive value for the high prevalence in the general population. In fact, more than 60% of men and 40% of women aged between 41 and 65 years, snore during the night [4, 9].

##### Witnessed Apnoeas and Restlessness:

For a good evaluation of patient with suspected OSAS, it is advantageous to interview the bed partner who can provide important information about characteristics and the frequency of breathing pauses during sleep. It is considered as a good diagnostic predictor of OSAS [4].

##### Nocturnal Choking or Gasping:

Some patients report waking at night with a choking sensation that passes after few seconds.

##### Other Nocturnal Symptoms:

Patients with OSAS less frequently report nicturia, excessive salivation, sleep fragmentation, arousals and gastro-esophageal reflux [10].

#### Daytime Symptoms

##### Excessive Daytime Sleepiness:

Obstructive sleep apnea is the most common sleep disorder that causes excessive daytime sleepiness (EDS). It leads to day time fatigue, decreased concentration, and an increased risk of traffic accidents.

##### Other Daytime Symptoms:

Many patients also report headache, memory impairment, and depression [10].

### Questionnaires

Currently, a limited number of screening tools are available to detect sleep disorder in adults. The Berlin questionnaire [11], which was designed to identify patients as being at ‘high’ or ‘low’ risk for OSAS, assesses the patient’s risk level based on approximately 11 questions addressing three symptom categories: snoring, sleepiness, and high blood pressure/weight. The Sleep Disorders Questionnaire (SDQ) is a valid tool to diagnose common sleep disorders, but due to its length (176 items) the author concluded that is not a practical instrument in primary settings [12]. The Pittsburgh Sleep Quality Index (PSQI) is a 24-item questionnaire useful for measuring changes in sleep quality over time in patients, alerting physicians for the need of further evaluation [13] Roth *et al.* purposed the Global Sleep Assessment Questionnaire (GSAQ) as a suitable screening tool for sleep disorders in general primary care population, although the author declared some study-limitations [11]. More recently some authors have purposed the SA-SDQ, a 12-item validated measure of sleep related breathing disorders, as a possible useful tool to screen epilepsy patients for OSAS, although appropriate cut-off points have not been established. In a recent work, we purposed the usefulness of a 7-items questionnaire, Rome Questionnaire (RQ), in identifying adult patients at risk of OSAS. The "RQ", together with BMI, seems to be a useful tool to make a selection of the patients at higher risk of moderate-severe OSAS, who need a prompt PSG evaluation [14].

### Polysomnography (PSG)

Sleep laboratory for overnight polysomnography (PSG) is the gold standard diagnostic tool for patients with suspected sleep apnea. This procedure includes continuous recordings of many physiologic data including airflow, chest/abdominal excursion, electroencephalography, electrooculography, electromyography, electrocardiography, and oxyhemoglobin saturation. However, PSG in the sleep laboratory is expensive, cumbersome, and not readily available in many geographic areas due to a growing demand for the procedure. Portable monitoring device based on a limited number of channels in an ambulatory setting such as the home could improve access to care and reduce costs. The American Sleep Disorders Association (ASDA) has classified diagnostic systems into four categories, based on the testing environment, technician attendance, and number of parameters recorded [15]. Level I is reserved for technician-attended in-laboratory PSG. Portable monitoring ranges from level II (unattended, full polysomnography) to level IV (single channel such as simple pulse oxymetry). ASDA level III is reserved for devices that monitor airflow, chest/abdominal excursion, heart/pulse rate, and oxyhemoglobin saturation. More recently proposed methods for quantification of OSAS are the cross-power index (CPI, the integral of the cross-spectrum modulus between concomitant fluctuations in systolic blood pressure and blood oxygen saturation), aimed to assess the cardiovascular impact of OSAS, and the peripheral arterial tonometry index (PAT), an indicator of acute arousal responses to OSA. The PAT signal measures the pulsatile finger arterial volume changes that are regulated by the adrenergic innervation of smooth muscles of finger vasculature, and thus reflects sympathetic nervous system activity. PAT may indirectly detect apnea/hypopnea events by identifying surges of sympathetic activation associated with the termination of these events. This information is further combined with heart rate and pulse oxymetry data that are considered by the automatic algorithm of the analysis system. This detects respiratory events and calculates the PAT RDI [16]. Due to the cost and limited availability of full polysomnography in a sleep lab, in practice screening for OSAS is mostly based on portable polysomnographic devices, often supported by the use of questionnaires [12].

### Pathophysiology

Normal sleep is distinguished as rapid eye movement (REM) sleep or no-synchronized sleep, which occurs cyclically and more frequently during the second half of the night, and non-REM sleep or synchronized, which is subclassified into four stages and the amount of sleep time. The four stages consist of drowsiness (stage 1), mild sleep (stage 2) and deep sleep (stage 3-4). Decreased heart rate, blood pressure and sympathetic nerve activity occur during non-REM sleep. REM sleep, instead, is characterized by increased electrical activity in the brain and sympathetic nerve outflow, moreover, it is connected to intermittent and abrupt changes in blood pressure and heart rate. In obstructive sleep apnea this homeostatic control is severely altered.

The critical pathophysiological feature of OSA is sleep-related collapse of the upper airway (UA) at the level of pharynx. Obstructive apneas and hypopneas occur because of intermittent complete and partial collapse of pharynx, respectively, during sleep. Pharyngeal collapse can occur at the end of expiration or at the beginning of inspiration. The collapse initially starts in the retropalatal/oropharyngeal areas in most (56–75%) OSAS patients (57, 127). This is followed by caudal extension of the collapse to the base of the tongue in 25–44% of patients (57, 127) and, finally, to the hypopharyngeal region in 0–33% of patients.

During normal sleep, some protective mechanisms assure partial patency of the upper airway. There are more than 20 skeletal muscles with tonic and phasic activity that constitute the pharyngeal mucosa, playing a role in airway dilatation and wall stiffening. In REM-sleep, this activity is reduced by muscle atonia. Anyway, muscle activity in the upper airway is modulated even by chemoreceptor responses to blood oxygen and carbon dioxide tensions, as well as local reflex mechanisms, such as the negative airway pressure associated with vigorous inspiration. A smaller caliber upper airway lumen during wakefulness and sleep has been noted in patients with OSAS versus normal controls. Volumetric MRI suggests that most of the responsible soft tissue, that in part can be explained by fat deposition, originates from the tongue and lateral pharyngeal walls and renders vulnerable to collapse the upper airway of persons with OSAS. As a possible compensatory mechanism for this anatomically compromised airway, patients with OSAS have increased electromyographic activity of the pharyngeal dilator muscles during wakefulness.

Recent literature suggests that other variables may play a role in OSAS. Inflammation and trauma to the upper airway due to snoring, vibration, extrinsic contraction or fatigue can lead to a denervation of pharyngeal dilator muscles or actual damage to the muscles themselves. These events render the muscles that are less able to response to negative airway pressure during an apneic event [17-19].

Although data are insufficient to clarify the role of nasal obstruction in the pathogenesis of OSAS, the nose may play a role in upper airways collapsibility. A nasal obstruction causes an increase in airflow resistance upstream from the collapsible portion of the pharynx. As a result, the degree of negative collapsing pressure is increased on inspiration, rendering the pharynx more collapsible.

These cycles of hypoxia and carbon-dioxide retention elicit oscillations in both cardiac parasympathetic and sympathetic nervous activity. At apnea termination, asphyxia triggers a brief arousal from sleep that abruptly increases sympathetic activity, and suppresses vagal tone, precipitating surges in blood pressure and heart rate. Intermittent hypoxia can induce oxygen-free-radical production and activate inflammatory pathways that impair vascular endothelial function. These adverse vascular effects, combined with increased sympathetic vasoconstrictor activity and inflammation, could predispose to hypertension and atherosclerosis. Cerebral blood flow declines significantly during obstructive apneas due to a decrease in cardiac output, and in the patients with flow-limiting lesions of the cerebral arteries, this can predispose to ischemic events.

A number of studies have reported an independent association of OSAS with several components of metabolic syndrome, particularly insulin resistance and abnormal lipid metabolism. OSAS-related factors that may contribute to metabolic dysregulation include increased sympathetic activity, sleep fragmentation, and intermittent hypoxia [20, 21].

### Therapeutical Options

#### Continuous Positive Airway Pressure and Oral Appliance

Continuous positive airway pressure (CPAP) represents the main treatment of OSAS that prevents airway collapse during respiration at night. Other strategies for treating OSAS include oral appliances [22]. In the same time, it is very useful in treating patients with sleep disordered breathing (SDB) to eliminate all possible contributing factors, including weight loss for patients who are obese and elimination of alcohol or sedative use, especially near bedtime and sleep postural changes, focusing on avoidance of sleeping in the supine position, weight loss, avoidance of alcohol and sedative hypnotics intake, and upper airway surgical procedures. Nasal continuous positive airway pressure (CPAP) is considered as an ideal treatment for treating OSAS, due to its being conservative and reversible, however, there is a poor rate of adherence in its long-term use.

For patients with mild and moderate OSAS patients who do not compliant or poorly compliant to nasal CPAP, oral appliance could be used. The rationale for oral appliances is to increase the posterior oropharyngeal airway space, therefore reducing upper airway collapsibility during sleep. Appliances are generally less successful with severe OSAS (AHI>50). Oral appliances can be classified as either tongue-retaining or mandibular-repositioning. [23, 24].

#### Medical Therapy

The exact target of pharmacological treatment for OSAS still remains to be defined, and currently, no widely accepted pharmacological treatment is available for OSAS [25]. Several potential drug candidates in OSA have already been investigated, but most of the trials have limited in size, and only few met the quality criteria applied in a drug study. Among the drugs, we have non-sedating tricyclic antidepressant protriptyline, the serotonin precursor L-tryptophan, the cholinesterase inhibitory agent physostigmine, theophylline, aminophylline, and acetazolamide [26-29].

Ideally, the medical treatment of OSAS should include not only the improvement of nocturnal breathing, but also of comorbid conditions such as hypertension, obesity, metabolic derangement, hormonal dysfunction, daytime sleepiness, and cognitive dysfunction [30].

Surgery can significantly complement those cases where CPAP is not tolerated (long-term compliance is not more than 50% due to several side effects), alleviate symptoms of daytime sleepiness, improve quality of life, and reduce the signs of sleep apnea recorded by polysomnography. The goal of surgical correction in the upper airway is to reduce the number and severity of obstructive events, when complete elimination of these events is not possible. Surgery for OSAS must be carried out taking the degree of obstructive apnea, and the place of greatest obstruction into account. Most OSAS patients have multilevel disease including nasal and retrolingual obstructions, and due to this reason the appropriate surgical treatment should be multileveled. In the last decade, a significant increase in publications on multilevel approach for OSAS patients illustrated the trend, away from single-level surgery. We have many surgical procedures from mini-invasive interventions for mild forms to more aggressive ones for severe OSAS cases [31].

The operative techniques included the following:

1. Nose—submucosal resection of septum and inferior turbinate, endoscopic sinus surgery, polypectomy, radiofrequency (RF) turbinate surgery, and nasal valve suspension.
2. Oropharynx—uvulopalatoplasty, tonsillectomy, transpalatal advancement pharyngoplasty, uvuloflap, uvulopalatal flap, extended uvulopalatal flap, laser-assisted uvuloplasty, RF palatal surgery, and Pillar implant technique.
3. Hypopharynx—maxillomandibular advancement, partial glossectomy, laser lingual tonsillectomy, genioglossus advancement, hyoid suspension, tongue base reduction with hypoepiglottoplasty, partial epiglottidectomy, hyoid suspension to the mandible, thyrohyoid advancement, tongue base suspension with the repose system, and RF tongue reduction [32].

## MAIN RISK FACTORS: THE GENETIC BASIS

Several previous reports have described OSAS occurring in genetically related subjects. Strohl *et al.* first described a family with multiple male relatives suffering from the symptoms of excessive daytime sleepiness and night time restlessness, from which three brothers had repetitive apneas during sleep [33].

Familial aggregation is generally explained by the fact that most risk factors involved in the pathophysiology of OSAS are, to a large extent, genetically determined.

The prevalence of OSAS is typically higher among certain ethnic groups, such as African-Americans, in which it appears to present at a younger age and may also be more severe than European-Americans. Redline *et al*., in a case-control family study, described the distributions of sleep-disordered breathing (SDB) in African-Americans and Caucasian. This study showed how African-Americans with SDB were younger than Caucasians with SDB (37.2 +/- 19.5 versus 45.6 +/- 18.7 yr, p < 0.01) and how, RDI level was higher in subjects < or = 25 years old in African-Americans [19]. Racial variations may be due to obesity that is more prevalent and more epidemic in African-Americans than in European-Americans. Anatomic features typical of each race like the increased upper airway soft tissue in African-Americans seems to play also an important role.

In a similar study, Baldwin *et al*., considered sleep related breathing disorders in Maori-Pacific Islanders and Europeans. Higher levels of severity parameters of OSAS (apnea-hypopnoea index, wake and minimum oxygen saturation, and apnea duration) were seen in both Maori and Pacific Islanders than Europeans [34].

The major risk factors for OSAS include obesity, ventilatory control abnormalities, and craniofacial dysmorphism (disproportionate craniofacial anatomy).72 [35]. To address the issue of genetic predisposition to OSAS, two basic designs are currently used: a systematic genome scan in multiplex families, and the study of candidate gene markers by case-control designs [36].

### Body Weight and Fat Distribution

There are several factors that increase the risk of OSAS development in general population. Among these, obesity is the most common clinical finding and it is present in more than 60% of the patients with OSAS [37]. Peppard *et al*. in a prospective study illustrate a relation between weight gain and OSA severity [38]. In particular, they found that a 10% weight gain is associated with an approximate 32% increase in the AHI and a 10% weight loss is associated with a 26% decrease in the AHI. However, the real causal pathways involved in the relationship between obesity and OSAS are not completely defined. Some studies proposed that hormonal mechanism may leads to the development of OSAS in the patients with obesity. In fact, adipose tissue produces leptin, a hormone that has an important effect on weight regulation by stimulation of hypothalamic satiety centers. O'Donnell *et al*. reported an important role for leptin in stimulating ventilation in the experiments using obese mouse mutated for the gene that encodes leptin [39]. In this way, a state of leptin resistance in obese subjects suggested by Considine *et al*. [40] may represents one of the causes of higher prevalence of OSAS in these patients.

Many studies have shown that there are some genes involved in the development of both obesity and OSA. Clearly, given the close relationship between obesity and OSAS, any genetic risk factor for obesity might also be considered a risk factor for OSAS.

A candidate gene is placed on chromosome 2, encoding for pro-opiomelanocortin (POMC). This is a pro-hormone that is converted in melanocyte-stimulating hormones (a-MSH) by proconvertase 1, and plays an important role to mediate the effects of leptin on appetite reduction, on increase of energy expenditure and on ventilatory drive. Another potential candidate gene is the COH1. It is located on the long arm of chromosome 8 and encodes a transmembrane protein that seems to be involved in vesicle-mediated sorting and intracellular transport [41]. Even if severe mutations in this gene causes the Cohen syndrome, characterized by mental retardation, microcephaly, prominent nose and short and upturned philtrum, truncal obesity, joint laxity, etc., only mild mutation in this one might be involved in a non-syndromic form of obesity and OSAS as suggested by Patel SR [42]. There are a number of studies showing the involvement of serotoninergic system in the development of both obesity and OSAS. This system is known to modulate mood, emotion, sleep, and appetite. In particular, serotonin stimulates the satiety center placed in arcuate nucleus and it excites adult upper airway dilator motor neurons [43]. For these reasons it is easy to understand how the reduction in serotoninergic activity leads to an increase in appetite and to a reduction in upper airway muscle tone, and in this way to obesity and OSAS. Miina Öhm *et al*., in a study conducted in Finnish population, suggest that a major locus affecting obesity phenotype would lie on chromosome Xq24 [44]. In 2003 Suviolahti *et al*. investigated the Xq22–24 region and found significant evidence of association between obesity and a polymorphism of SLC6A14 gene. It encodes Na- and Cl-dependent membrane protein involved in the transport of tryptophan, the precursor of serotonin, across the plasma membrane into CNS [45]. This association was also confirmed in a French study conducted by Durand *et al*. [46].

Body fat distribution plays an important role in the development of OSAS. It is easy to understand that the fat deposition in upper airway lumen may increase its collapsibility. Shinohara *et al*. suggest that visceral obesity is associated more often with OSAS rather than other forms of obesity [47]. They consider that the mass weight of visceral fat may cause an increase in the activities of the inspiratory muscles by restricting the motion of the diaphragm. This results in increased inspiratory negative airway pressure leading to collapse in the upper airway. BMI (Body Mass Index) is the most common parameter to describe obesity worldwilde and many studies suggest the strength of this index to predict OSAS [4, 48]. There are some available anthropomorphic measurements to predict the presence of OSAS and its severity. Dixon *et al*. consider the neck circumference as the best single measure for predicting OSAS. For Schafer *et al*. the percentage of body fat, measured by BIA (Bioelectrical Impedance Assay), and BMI are the only significant predictors for OSAS. Ögretmenoglu *et al*. considered the percentage of body fat and BMI as important parameters for predicting OSAS and also proved the relationship between visceral abdominal fat, measured by CT, and AHI [48]. It must be considered that not all forms of obesity play the same role in the development of OSAS. In particular, fat deposition in the neck and the typical fat deposition pattern of male gender (in the subcutaneous regions of torso and abdomen) could be considered as predictors of OSAS [49]. In 2000, Katzmarzyk *et al*. estimated the level of familial similarity in anthropometric indicators of fatness and fat distribution and found a heritabilities of 29–48% for three indicators of fat distribution: BMI-adjusted waist circumference and ratio of trunk to extremity skinfold ratio [50]. These findings suggest the role of genes in explaining at least part of the heritability.

### Craniofacial Morphology

Several studies demonstrated the relationship between many morphological factors and OSAS. About the skeletal morphology, it was found that SNA and SNB values were significantly reduced in patients with OSAS, suggesting a link between retrognathia, micrognathia and this disease [51]. Also an increase in soft palate thickness, length and area, in tongue’s length and area and in intermaxillay space length, leads to the reduction of size of the upper airway and so to higher risk of OSAS [51]. Riha *et al*. also found that a lower-set hyoid bone in relation to the mandibular plane is significantly associated with a diagnosis of OSAS [52]. In fact, the hyoid bone, where the attachments of the lingual musculature are located, pulls down the tongue leading a narrowing of the pharyngeal airway.

Inherited abnormalities of craniofacial structure appear to explain at least a portion of the familial clustering of OSAS, and the genetic basis is suggested by twin and family studies.

Mathur *et al.* revealed that relatives of OSAS patients showed total narrow pharyngeal volumes and glottic cross-sectional areas, retropositioned maxillae and mandibles, and longer soft palates as compared with the relatives of controls [53].

Guilleminault *et al.* found that OSAS patients have some mild craniofacial disproportions, and in 1986, they published that infants with apparently life-threatening events and sleep apnea typically have family members with obstructive sleep apneas and that small upper airways, indicated by cephalometric roentgenograms and volume CT scans, were a common familial feature.

In a large study published in 1995 Guilleminault *et al.* showed that a disproportionate craniofacial anatomy was common in familial groups with OSAS, concluding that craniofacial familial features can be a strong indicator of risk for the development of OSAS [54].

There are many genetic syndromes associated with a number of craniofacial dysmorphisms and it could be considered another link between genes and OSAS. One of these is Down’s syndrome in which OSAS is very common. Marfan syndrome, carried by a gene called FBN1, which encodes a connective protein fibrillin-1, may also causes craniofacial dysmorphisms and upper airway connective tissue laxity, and in this way OSAS [55]. Craniofacial abnormalities such as retrognathia and micrognathia were noticed in mice deficient in endothelin-1 [56], with mutations of the retinoic acid receptors (RARs) [57] and of transforming growth factor-beta2 (TGFbeta2) [58].

### Respiratory Control System

The presence of ventilatory instability in OSAS patients was first noted when periodic breathing accompanied by central apneas, occurred after tracheostomy [59].

Induction of periodic breathing during sleep by hypoxia, but only in the presence of inspiratory resistive loading, can induce UA obstruction in normal subjects [60].

It is possible that ventilatory patterns are modified during hypercapnia or hypoxemia through chemoreflexes control.

Peripheral chemo receptors, the most important of which are located in the carotid bodies of the internal carotid arteries, primarily respond to blood oxygen tension, while brain-stem central chemo receptors are most sensitive to carbon dioxide and acid-base balance. In healthy persons, the chemoreceptor response is reduced during sleep as compared with wakefulness, and it leads to modest changes in blood gas tensions (increase in partial pressure of carbon dioxide of 2 to 6 mmHg and decrease in oxygen saturation of up to 2%). Some studies have shown that patients with OSAS have a greater peripheral chemoreflex sensitivity, producing an increased ventilatory response to hypoxemia. Enhanced chemoreflex sensitivity in OSAS may explain the exaggerated sympathetic response during hypoxemic episodes. In healthy persons, there are interactions between the chemoreflex and baroreflex responses. The baroreflex attenuates ventilatory, sympathetic, and bradycardic responses to peripheral chemoreflex excitation. Baroreflex dysfunction causes a disinhibition of chemoreflex responses, it can occur in hypertension or heart failure and may have relevance when cardiovascular disease coexists with OSAS [3].

A hallmark of OSAS is a marked reduction in pleural pressure, because of respiratory efforts against a restricted or collapsed airway.

Some research groups have noted that potentially inherited abnormalities of ventilatory control may predispose to obstructive or central sleep apnea by influencing ventilation during sleep and increasing the propensity to upper airway collapse. Altered ventilatory drive also may precipitate apnea by promoting ventilatory control instability and, subsequently, periodic breathing. Ventilatory control instability could result from either blunted or augmented chemosensitivity.

An inherited basis for ventilatory responsiveness to hypoxemia or hypercapnia has been suggested by the findings from several human studies. Troubles in ventilatory responses to hypoxia or hypercapnia have been described in the first degree relatives of the people with various pulmonary diseases or syndromes. Several twin studies have demonstrated similarities in ventilatory responses to hypoxia or hyperoxia to be greater in MZ than in DZ twins, concluding a genetic basis for the chemoresponse to blood oxygen saturation [61].

Heritability for chemoresponsivity to oxygen saturation levels vary between approximately 30% and 75%, suggesting a substantial contribution of inheritance to this trait. A greater reduction of ventilatory responses to progressive eucapnic hypoxia during wakefulness is observed in the members of OSAS families as compared to members of control families. Moreover, impairment in load compensation was suggested by the finding of a significantly greater increase in ventilatory impedance with inspiratory resistive loading in OSAS family members versus control subjects [62]. These data point out that the familial aggregation of OSAS may be based on inherited abnormalities in ventilatory control, probably related to chemoregulation and/or load compensation. In genetically susceptible individuals, the upper airway appears sensitive to excess collapsibility during conditions of mild inspiratory loading. Some clues regarding the underlying genetics of ventilatory control abnormalities in OSAS may be gleaned from studies of children with congenital central hypoventilation (CCH) syndromes. These are children with frequent apneas and daytime hypoventilation, attributable to severe chemoregulatory dysfunction, because of profound blunting of the hypercapnic and hypoxic ventilatory responses. In some cases, developmental abnormalities of the brainstem or cerebral cortex have been found. In the absence of secondary causes, they are labeled as “idiopathic congenital central hypoventilation”. Familiarity in CCH has been described and several analyses suppose that the disorder can be explained by either multifactorial threshold or major locus models. Hirschsprung’s disease, congenital disorder with intestinal dysmotility and absence of myenteric and submucosal ganglia in the distal bowel, may occur in as many as 50% of cases of CCH [63].

In children with Hirschsprung’s disease, mutations of both the RET proto-oncogene have been described, encoding a receptor tyrosine kinase that is thought to be involved in neural crest migration and proliferation, and RET ligand, glial cell line-derived neurotrophic factor (GDNF) [64].

So, it is possible that CCH syndromes may be sometimes caused by abnormalities in migration of neural crest cells to central respiratory control centers. Moreover, other genes involved in the endothelin signaling pathway (endothelin B receptor gene, EDNRB and endothelin 3 gene, EDN3) have been implicated in Hirschsprung’s disease and could be considered as candidate genes for CCH syndromes and sleep apnea. Endothelin-1 (ET-1), a potent vasoactive peptide, may participate the control of ventilation. In a knockout mouse model, absence of ET-1 results in respiratory failure, ventilatory control abnormalities, craniofacial abnormalities and hypertension; characteristics remarkably similar to traits found in OSAS [56].

A European group has demonstrated that when the zinc finger protein Krox-20 is deleted, mice show slow respiratory frequencies and long apneas. Another group of researchers has seen a reduced survival of neurons in the nodose-petrosal ganglion, when they study knockout mouse model of brain derived neurotrophic factor (BDNF). Anyway, homozygous mice for BDNF alterations demonstrate irregular and depressed ventilation, spontaneous apneas and abnormalities in chemoregulation, specifically related to hyperoxia but not to hypercapnia [65].

Together these data show the complexity of the respiratory control system, and that a number of genes, important in different native regulatory functions, may influence ventilatory phenotypes.

Other candidate genes include a family of genes that encode neuroreceptors (e.g., glycine receptor, glutamate receptor), and genes that influence the post-natal development of the lung (e.g. basic fibroblast growth factor, bFGF). Some groups have examined breathing frequency during hypoxia and proposed a two-gene model, involving genes that are different from those that determine baseline frequency [66]. Finally, another probable link between respiratory control and the 8q22 chromosomal region has been observed. It contains three genes for carbonic anhydrase (CA) isoenzymes: CA1, CA2, and CA3. Several animal and human studies are going to confirm the roles of CA in modulating respiratory control, and the role of CA inhibitors as potential treatment for conditions as respiratory instability, sleep periodic breathing, and OSA [67].

## CONCLUSION

There is a continuous interaction in the structural development of the upper airway region, between genetic and environmental factors. Clinical and epidemiological studies show that OSAS is a multifactorial and complex disease with a strong genetic basis. Many genes are involved, from genes that induce migration of specific cellular groups to genes leading to the structural organization The identification of genes implicated through human and animal models in OSAS would help to elucidate the OSAS pathogenetic processes, still largely obscured.
